# 

**Published:** 1854-07

**Authors:** 


### RECEIPTS DURING JUNE AND JULY.

### ILLINOIS

| | | | |
|---|---|---|---|
| Dr. E Dickinson | 4 00 | Dr E W Grow, | 6 00 |
| J Higgins, | 2 00 | J R Abell, | 2 00 |
| R Morris, | 2 00 | W McKnight, | 2 00 |
| Newell, | 2 00 | C D Pearson, | 4 00 |
| T A Graham, | 2 00 | O W Knott. | 2 00 |
| J H Robinson, | 2 00 | J G Tilden, | 2 00 |
| G M Fox, | 2 00 | C N Irwin. | 2 00 |
| T Seeley, | 2 00 | N D Thomas, | 1 00 |
| F R Payne, | 2 00 | Smith & Secrest, | 2 00 |
| J S Bullock | 2 00 | R A Saunders, | 3 00 |
| A Ballou, | 1 00 | Mack, | 1 00 |
| Hay. | 2 00 | Hall, | 2 00 |
| Hanley, | 2 00 | L Osborn, | 2 00 |
| W F Coleman, | 3 00 | Kirwan, | 2 00 |
| Danforth, | 2 00 | Jenks, | 2 00 |
| Hitchcock, | 1 50 | F B Haller, | 2 00 |
| Little & Lewis | 2 00 | McKinney, | 2 00 |

### INDIANA.

| | | | |
|---|---|---|---|
| Dr A Foster, | 1 00 | Dr C D Pearson | 4 00 |
| T A Graham, | 2 00 | J Worden, | 2 00 |
| Hendrick, | 2 00 | Glassbrook, | 2 00 |
| Thompson, | 9 00 | Maxwell. | 2 00 |
| Brenton, | 2 00 | Davenport, | 6 00 |

### WISCONSIN

| | | | |
|---|---|---|---|
| Dr J Phillips | 2 00 | Dr Johnson, | 2 00 |
| E G Little | 2 00 | Adams, | 2 00 |
| Coleman, | 2 00 | | |

### MICHIGAN.

| | | | |
|---|---|---|---|
| Dr McAvey, | 2 00 | Dr Chamberlain, | 2 00 |
| McArthur | 4 00 | | |

| | |
|---|---|
| Dr A W Dewey, Ohio, | 2 00 |
| Dr F Brady, Iowa, | 2 00 |
| Dr Elliott, do. | 2 00 |

